# Evaluation of the Effect of a Continuous Treatment: A Machine Learning Approach with an Application to Treatment for Traumatic Brain Injury

**DOI:** 10.1002/hec.3189

**Published:** 2015-06-08

**Authors:** Noémi Kreif, Richard Grieve, Iván Díaz, David Harrison

**Affiliations:** ^1^ Department of Health Services Research and PolicyLondon School of Hygiene & Tropical MedicineLondonUK; ^2^ Department of BiostatisticsJohns Hopkins Bloomberg School of Public HealthBaltimoreMDUSA; ^3^ Intensive Care National Audit & Research CentreLondonUK

**Keywords:** programme evaluation, generalised propensity score, machine learning

## Abstract

For a continuous treatment, the generalised propensity score (GPS) is defined as the conditional density of the treatment, given covariates. GPS adjustment may be implemented by including it as a covariate in an outcome regression. Here, the unbiased estimation of the dose–response function assumes correct specification of both the GPS and the outcome‐treatment relationship. This paper introduces a machine learning method, the ‘Super Learner’, to address model selection in this context. In the two‐stage estimation approach proposed, the Super Learner selects a GPS and then a dose–response function conditional on the GPS, as the convex combination of candidate prediction algorithms. We compare this approach with parametric implementations of the GPS and to regression methods. We contrast the methods in the Risk Adjustment in Neurocritical care cohort study, in which we estimate the marginal effects of increasing transfer time from emergency departments to specialised neuroscience centres, for patients with acute traumatic brain injury. With parametric models for the outcome, we find that dose–response curves differ according to choice of specification. With the Super Learner approach to both regression and the GPS, we find that transfer time does not have a statistically significant marginal effect on the outcomes. © 2015 The Authors. *Health Economics* Published by John Wiley & Sons Ltd.

## 1 Introduction

Public policymakers may be interested in evaluations that estimate the causal effects of treatments measured on a continuous scale. For example, evaluations may attempt to estimate the marginal causal effects of alternative financial incentives for health care providers, different levels of taxation on addictive substances and increasing doses of a new pharmaceutical. In such settings, the expected outcome at alternative levels of the treatment can be reported from the estimated dose–response function. When estimating such dose–response functions, observed confounding can be adjusted with regression, which models the outcome as a function of the treatment variable and observed covariates. Regression approaches can be simple, for example, ordinary least squares regression, or can take more flexible forms such as fractional polynomials (Royston and Altman, 1994; Royston *et al.*, 2006) or generalised additive models. If the outcome regression model is misspecified, estimates of the dose–response function will be be biased.

The generalised propensity score (GPS) approach has been proposed (Imbens, 2000; Hirano and Imbens, 2004) as an alternative to regression for evaluating continuous or multi‐valued treatments. Both the standard regression method and the GPS approach assume ‘unconfoundedness’ or that adjusting for observed covariates is sufficient to achieve independence between potential outcomes and the treatment level received. As with the propensity score for binary treatments, rather than adjusting for a vector of covariates, the GPS adjusts for a one‐dimensional score, the conditional density of treatment, given baseline covariates. Hirano and Imbens (2004) proposed a regression model of the outcome as function of just two covariates, the treatment and the GPS. This approach has been followed by subsequent empirical work (Bia and Mattei, 2008; Bia *et al.*, 2011; Kluve *et al.*, 2012). Alternative implementations of the GPS include a kernel weighting approach (Flores *et al.*, 2012) and using the GPS for inverse weighting of marginal structural models (Robins *et al.*, 2000). Imai and Van Dyk (2004) introduced a similar concept, the ‘propensity function’ for estimating the average treatment effect in strata defined by the propensity function. Yang *et al.* (2014) develop methods of matching and classification on the GPS, to estimate the effects of multi‐valued treatments. This paper considers further the approach by Hirano and Imbens (2004), proposed for continuous treatments.

A general challenge with the GPS approaches proposed is to correctly specify the outcome and GPS models. For the outcome model, previous studies have proposed fully parametric models with polynomials (Hirano and Imbens, 2004; Bia and Mattei, 2008) or semi‐parametric approaches with regression splines (Kluve *et al.*, 2012; Bia *et al.*, 2011). Bia *et al.* (2011) compared parametric and semi‐parametric estimators of the outcome model, and found that the estimated dose–response function was robust to the choice of semi‐parametric approach, but it was sensitive to parametric specification. For the GPS estimation, most studies have assumed a normal or lognormal distribution and suggested that model specification is assessed with indirect tests of the balancing property (Hirano and Imbens, 2004; Imai and Van Dyk, 2004). Evidence from evaluations of binary treatments is that misspecification of propensity score and outcome models can lead to severe bias (Kang and Schafer, 2007). However, no studies have investigated the effects of model misspecification of the GPS on the estimated dose–response function, nor compared the GPS and regression approaches.

We propose a framework for estimating the marginal causal effects of continuous treatments, which can mitigate the problem of model misspecification. Here, both the GPS and the outcome model are selected by a machine learning algorithm, the Super Learner. The Super Learner aims to construct an improved estimator, by finding the optimal weighted combination of several prediction algorithms (van der Laan and Dudoit, 2003; van der Laan *et al.*, 2004; van der Laan *et al.*, 2007). It has been extensively used for estimating the propensity score and the outcome model, for estimating treatment effects (Petersen *et al.*, 2008; van der Laan and Rose, 2011; Eliseeva *et al.*, 2013; van der Laan and Luedtke, 2014). The Super Learner has been shown to reduce bias from model misspecification in binary treatment settings (Porter *et al.*, 2011; Kreif *et al.*, 2014; Pirracchio *et al.*, 2015b), but has not been previously combined with the GPS.

The aim of this paper is to consider the Super Learner for estimating dose–response curves. We use the Super Learner to estimate the GPS and to model the relationship of the outcome with treatment and the GPS. We compare this method to estimating the outcome model with parametric models. Furthermore, we consider the standard, multivariable regression approach to estimate the dose–response, and again, we compare parametric models to using the Super Learner for model selection.

We demonstrate an important advantage of using the Super Learner, compared with the informal process of selecting the best fitting model, often performed in applied research. The Super Learner, by explicitly considering the performance of the underlying algorithms, allows the process of model selection to be made transparent.

The methods are contrasted in an empirical example, in which we evaluate the marginal causal effect of increasing the transfer time from an emergency department to specialist neuroscience centres for critically ill patients with traumatic brain injury (TBI). We use data from the Risk Adjustment in Neurocritical Care (RAIN) cohort study (Harrison *et al.*, 2013).

The paper is organised as follows. Section 2 briefly describes the potential outcomes framework for dose–response functions and reviews the regression and GPS approaches to estimating dose–response curves. We then introduce the Super Learner for estimating dose–response functions. Section 3 describes the methods used in our empirical application and presents the results. Section 4 concludes and discusses limitations and further research.

## 2 Methods

### 2.1 Dose–response functions

Following Hirano and Imbens (2004), we define dose–response functions in the potential outcomes framework (Rubin, 2005). Let *i* = 1 to *n* be randomly sampled units. The continuous treatment of interest can take values in *t*∈*τ*, where *τ* is an interval [*t*_0_,*t*_1_]. For each unit, let *Y*_*i*_(*t*):*t*∈*τ* be a set of potential outcomes (the unit‐level dose–response function), each corresponding to the outcome in a hypothetical world in which *T* = *t* is set deterministically. The parameters of interest are first, the average dose–response function, *μ*(*t*) = *E*[*Y*_*i*_(*t*)], second, the marginal treatment effect function (Bia and Mattei, 2008; Bia *et al.*, 2011), capturing the effect of increasing the level of treatment on the expected potential outcome: (*E*[*Y*_*i*_(*t*)] − *E*[*Y*_*i*_(*t*−Δ*t*)])/Δ*t*. For example, with Δ*t* = 1, the parameter captures the incremental change in the outcome, for a unit change in the level of treatment.

For each unit, we observe a vector of covariates *X*_*i*_, the level of treatment received, *T*_*i*_∈[*t*_0_,*t*_1_], and the observed outcome, which corresponds to the potential outcome under the level of treatment received, *Y*_*i*_=*Y*_*i*_(*T*_*i*_). The weak unconfoundedness assumption requires the independence of the potential outcome and the observed level of treatment for each value of treatment: *Y*(*t*)⟂*T*|*X* for all *t*∈*τ*. The consistency assumption requires that the observed outcome corresponds to the potential outcome under the treatment level received, formally, that *T* = *t* implies *Y*(*t*) = *Y*. The positivity assumption requires that the conditional density of the treatment is non‐negative for any covariate values, *P*(*r*(*t*,*x*) > 0) = 1, where we define *r*(*t*,*x*) ≡ *f*_*T*|*X*_(*t*|*x*) as the conditional density of treatment given covariates. Under these assumptions, the conditional expectation of the outcome under each treatment level can be estimated using the observed outcomes: *E*[*Y*(*t*)|*X* = *x*] = *E*[*Y*|*T* = *t*,*X* = *x*] and the average dose–response curve can be estimated by taking an expectation over *X*,*E*[*E*[*Y*|*T* = *t*,*X* = *x*]]. Regression estimators aim to model the observed outcome as a function of the treatment level *T* and covariates *X*:*Q*(*t*,*x*) = *E*[*Y*|*T* = *t*,*X* = *x*]. The average dose–response is defined as *μ*(*t*) = *E*[*Q*(*t*,*X*)].

### 2.2 The generalised propensity score method

The GPS is defined as the random variable *R* = *r*(*T*,*X*), the conditional density evaluated at the treatment level received and the covariates observed. The key feature of the GPS is its balancing property, informally stating that, within strata of the same value of the GPS evaluated at a given treatment level, *r*(*t*,*X*), the probability that the treatment received equals this treatment level, *T* = *t*, does not depend on the values of the covariates. This property, combined with the weak unconfoundedness assumption, implies that the GPS can be used to eliminate any bias associated with differences in the observed covariates amongst groups of units with different levels of treatment. It also implies that the counterfactual expectation *E*[*Y*(*t*)] is identified as *μ*(*t*) = *E*[*β*(*t*,*r*(*t*,*X*))], where *β*(*t*,*r*) = *E*[*Y*|*T* = *t*,*R* = *r*] is the conditional expectation of the observed outcome given the treatment level and the GPS.

The estimation of dose–response curves using the GPS involves two stages. First, the conditional density of the treatment is estimated, and the GPS is evaluated, at the level of treatment actually received, $\hat{R}=\hat{r}(T,X)$, and for the potential levels of treatment, ${\hat{R}}^{t}=\hat{r}(t,X)$. In the second stage, the conditional expectation of the outcome is estimated, given the treatment level and the GPS, *E*[*Y*|*T* = *t*,*R* = *r*]. For example, assuming that the outcome is a quadratic function of the treatment level and the GPS: $E[Y|T=t,\hat{R}=r]=\alpha_{0}+\alpha_{1}t+\alpha_{2}t^{2}+\alpha_{3}r+\alpha_{4}r^{2}+\alpha_{5}\mathrm{rt}$. Then the average dose–response function is estimated at each treatment level of interest, by averaging the previously estimated conditional expectation over $r={\hat{R}}^{t}$. This involves taking the estimated regression coefficients, and obtaining predictions for each unit, by plugging in the treatment level of interest, and the GPS evaluated at the treatment level of interest, for example: $\hat{\mu }(t)={\hat{\alpha }}_{0}+{\hat{\alpha }}_{1}t+{\hat{\alpha }}_{2}t^{2}+{\hat{\alpha }}_{3}Ê({\hat{R}}^{t})+{\hat{\alpha }}_{4}Ê({\hat{R}}^{t^{2}})+{\hat{\alpha }}_{5}\mathrm{tÊ}({\hat{R}}^{t})$, where Ê denotes the empirical mean operator. Of course, models of this type are often misspecified, which motivates using a more flexible regression approach.

### 2.3 The Super Learner in estimating the dose–response

The previous sections outlined the general approach for identifying and estimating the dose–response functions using the regression and the GPS approaches. For both methods, an outcome model must be specified, *Q*(*t*,*x*) for the regression, and *β*(*t*,*r*) for the GPS method. For the GPS approach, the conditional density of the treatment received also needs to be specified. We propose to use the Super Learner (van der Laan and Dudoit, 2003; van der Laan *et al.*, 2007; Polley and van der Laan, 2010), an ensemble prediction algorithm, which exploits machine learning, to estimate the outcome and the GPS models.

In general, machine learning covers a wide range of classification and prediction algorithms. Unlike approaches that assume a fixed statistical model, for example, a generalised linear model (GLM), machine learning aims to extract the relationship between the endpoint and covariates through a learning algorithm (Lee *et al.*, 2010). Machine learning approaches can reduce bias from misspecification of the outcome model (Austin, 2012), and the propensity score, in the context of binary treatments (Lee *et al.*, 2010).

The Super Learner is a stacked prediction method that uses cross‐validation to select the optimal convex combination of a list of prediction algorithms, where optimality is defined with respect to a pre‐specified loss function. The choice of the loss function is driven by the prediction problem and characteristics of the data: for predicting the conditional mean, the mean squared error (MSE) is a natural loss function, while for estimating the conditional density, the negative log likelihood is recommended (van der Laan and Dudoit, 2003; van der Laan *et al.*, 2004).

The Super Learner is asymptotically optimal in that it chooses the candidate with the smallest true expected loss (the so‐called risk) (van der Laan *et al.*, 2004). The range of prediction algorithms is pre‐selected by the user, potentially including parametric and non‐parametric regression models, and more general prediction algorithms. Asymptotically, the Super Learner algorithm performs as well as the best possible combination of the candidate estimators (van der Laan and Dudoit, 2003). The following sections provide a brief overview of the algorithm, first to estimate the outcome regression, then to estimate the conditional density. For details of the theory, see van der Laan and Dudoit (2003) and van der Laan *et al.* (2004, 2007), and for applications in prediction, see Polley and van der Laan (2010) and Rose (2013).

#### 2.3.1 The Super Learner to estimate the outcome regression

For predicting the conditional mean of the outcome, we choose the cross‐validated MSE as the loss function for the Super Learner (van der Laan and Dudoit, 2003; van der Laan *et al.*, 2004) 1. Let ${\hat{Q}}_{j}(w):j=1,\ldots ,K$ denote a list of regression estimators for the conditional expectation of the outcome, *Q*(*w*) = *E*(*Y*|*W* = *w*). For the regression approach, the vector *w* includes (*x*,*t*), and in the case of the GPS method, it consists of (*r*,*t*).^2^

We partition the sample in *V* validation samples *S*_*v*_:*v* = 1,…,*V*. Let $Q_{j,\bar{v}}$ be the *j*‐th candidate trained in the sample excluding validation sample *v*. We apply each estimator in the training sample and use the estimated model to predict the outcomes in the validation sample. We estimate the risk, the cross‐validated MSE of each estimator as

$$L({\hat{Q}}_{j})=\frac{1}{V}\overset{V}{\underset{v=1}{\sum }}\frac{1}{n_{v}}\underset{i\in S_{v}}{\sum }{\left(Y_{i}-{\hat{Q}}_{j,\bar{v}}(w_{i})\right)}^{2}.$$

The Super Learner estimator is a convex combinations of the list of estimators, which minimises the risk. Formally, we consider the candidate Super Learner estimators as

$${\hat{Q}}_{\alpha }(w)=\underset{j}{\sum }\alpha_{j}{\hat{Q}}_{j}(w),$$

and choose *α* to minimise $L({\hat{Q}}_{\alpha })$ with the constraint that *α*_*j*_≥0 and $\underset{j}{\sum }\alpha_{j}=1$. The cross‐validated MSE of this estimator (the ‘convex super learner’) can be obtained by repeating the cross validation procedure from the first step, after adding the convex Super Learner to the list of candidate estimators (Polley and van der Laan, 2013).

#### 2.3.2 The Super Learner to estimate the generalised propensity score

Here, we modify the aforementioned algorithm, to select an estimator of the conditional density of the treatment given covariates, by selecting a convex combination of candidate estimators, that minimises the loss function defined as the cross‐validated negative log likelihood (van der Laan and Dudoit, 2003; van der Laan *et al.*, 2004). Let ${\hat{r}}_{j}(t,x):j=1,\ldots ,K$ denote a list of GPS candidate estimators. For example, the conditional density can be derived from modelling *T* as a random variable following a normal or gamma distribution. Candidate estimators can also include variations of these with different higher order terms in the linear predictor of the mean.

We partition the sample in *V* cross‐validation splits. Let $r_{j,\bar{v}}$ be the *j*‐th candidate trained in the sample excluding split *v*. We compute the cross‐validated risk of each estimator as

$$L({\hat{r}}_{j})=\frac{1}{V}\overset{V}{\underset{v=1}{\sum }}\frac{1}{n_{v}}\underset{i\in v}{\sum }-\log{\hat{r}}_{j,\bar{v}}(t_{i},x_{i})$$

We consider estimator candidates of the form

$${\hat{r}}_{\alpha }(t,x)=\underset{j}{\sum }\alpha_{j}{\hat{r}}_{j}(t,x)$$

We choose *α* to minimise $L({\hat{r}}_{\alpha })$ with the constraint that *α*_*j*_≥0 and $\underset{j}{\sum }\alpha_{j}=1$. We use the ‘rsolnp’ package in R to perform the optimisation (Ghalanos and Theussl, 2012).

#### 2.3.3 The proposed estimation approaches

We compare two different Super Learner‐based estimators of the dose–response curve. First, in a two‐stage process, the Super Learner estimates the GPS and then uses the estimated GPS to select a model for the outcome, as a function of the treatment variable and the estimated GPS. The second estimator is a standard regression adjustment approach whereby the Super Learner estimates the outcome model as a function of the treatment and covariates, and the predictions are averaged across covariates at each level of the treatment. We describe the candidate algorithms of the Super Learner in more detail in the next sections.

## 3 Empirical Example

### 3.1 The RAIN cohort study

Acute TBI imposes a large burden in terms of cost and mortality (Harrison *et al.*, 2013). An important public policy question is how best to manage critically ill adult patients following an acute TBI. In particular, there are large local variations in the time to transferring patients from initial hospital presentation to arrival at a specialised neuroscience centre. The primary aim of the RAIN study was to validate risk prediction models for acute TBI, for use in evaluating the relative costs and outcomes of alternative locations of neurocritical care in the National Health Service (Harrison *et al.*, 2013). A total of 67 critical care units participated in the RAIN study, with 3626 admitted patients providing a representative sample of patients receiving critical care following acute TBI in UK. The data followed a cross‐classified structure, whereby patients admitted to a particular hospital could be transferred to one of several specialist neuroscience centres within that geographical region 3.

An important research question the RAIN study aimed to answer was whether adult patients with acute TBI without an acute neurosurgical lesion benefit from an early decision to transfer to a neuroscience centre. The clinical literature is not conclusive about the benefits of early transfer: while some studies suggest that in patients for whom neurosurgery is not indicated (Bullock *et al.*, 2006), the risks from early transfer and subsequent aggressive protocols of care may be substantial, an alternative view is that an early decision to keep the patient within the non‐neuroscience centre can lead to delayed transfers, for example, if a critical lesion develops subsequently, with potentially higher risks (Shafi *et al.*, 2008). The RAIN study compared early (within 18h of hospital presentation) transfer to a neuroscience centre with no or late (after 24h) transfer, for patients who initially present at a non‐neuroscience centre and do not require neurosurgery, and found that at 6months, patients in the early transfer group had significantly lower mortality, however higher total costs.

This raised a further research question of how, once the decision to transfer early had been made, variations in transfer time affect expected costs and mortality. Exogenous variation in transfer time is expected because of patient characteristics, local variations in management and delays due to logistics. By investigating the causal effect of transfer time on outcomes, important insights may be gained for subsequent guideline development, for example, on the benefits of reducing logistical barriers to shorten transfer times. We aim to address this research question by estimating the dose–response relationship between transfer time and 6‐month mortality, and transfer time and 6months costs.

### 3.2 Data

We define the population of interest as adult patients with acute TBI who presented outside of a neuroscience centre, did not require neurosurgery and were transferred to a neuroscience centre within 24h. Transfer time is the time in hours between admission to the emergency department at the presenting hospital and admission to the specialist neuroscience centre. This definition, based on consultation with a panel of clinicians, reflects that a transfer more than 24h after hospital presentation implies a decision to delay transfer, rather than an intended early transfer delayed by logistics. Transfer time consists of the time spent at the emergency department at an intermediate location such as a different ward of the admitting hospital, or a different hospital that is not a neuroscience centre, and time spent in transit between locations.

We aim to control for all variables, which are potential confounders in the relationship between transfer time and mortality, and transfer time and costs, that is, variables that might influence transfer time and also affect these outcomes. We observe the prognostic variables that might influence the clinicians' decision on when to transfer, measured after the patient has been stabilised but before the decision has been made to transfer the patient. We use the variables from the International Mission for Prognosis and Analysis of Clinical Trials in TBI (IMPACT) lab model (Steyerberg *et al.*, 2008), selected based on cross‐validation in the RAIN study, including clinical factors measured after stabilising the patient (hypoxia, hypotension, motor score, Glasgow Coma Score (GCS), pupil reactivity, Marshall computed tomography classification, presence of traumatic subarachnoid haemorrhage, presence of extradural haematoma and lab measurements). Informed by clinical opinion, we include further important potential confounders: the presence of major extracranial injury, last pre‐sedation GCS, variables indicating suspected or confirmed intoxication, age and gender. The IMPACT‐predicted mortality is a composite score expressing the probability of mortality based on the IMPACT lab risk prediction model and aims to capture the baseline severity of patients. The descriptive statistics on the key potential confounders, as well as the outcomes, are presented in Table 2.

Mortality is measured as all cause mortality at 6‐month follow‐up. Costs up to 6months are measured by considering resource use from the first critical care admission following the TBI and readmissions within 6months. Each resource use item was combined with the appropriate unit cost to report a cost per patient for each cost category in 2010–11 prices.

Missing outcome and covariate data have been addressed with multiple imputation using chained equations (five datasets) (Buuren and Groothuis‐Oudshoorn, 2011). In order to demonstrate the approach, we present results on the first of the imputed datasets. Given the cross classified nature of the data, this re‐analysis followed the approach taken in the original study (Harrison *et al.*, 2013) and did not allow for potential clustering.

### 3.3 Implementation

#### 3.3.1 Candidate algorithms in the Super Learner

For both the estimation of the GPS and the outcome, we specify the Super Learner libraries with the intention of increasing the flexibility of the candidate prediction algorithms, beyond considering the usual parametric models. We also take into account knowledge of the distribution of the treatment variable and the outcomes.

We consider a range of candidate estimators for estimating the GPS, including normal and gamma models, the latter capturing the skewed distribution of the treatment variable, with higher order terms and interactions reflecting clinical opinion about potential nonlinear effects of covariates on the treatment assignment (see the specifications in Table 1). We use the Super Learner to select the best convex combination of these models, and estimate the GPS, which we use throughout the analysis.

**Table 1:** Specifications of candidate algorithms in Super Learner

GPS estimation						
Candidate	Error dist	Link fn	Linear pred			
Norm 1	normal	identity	*W*,*A*			
Norm 2	normal	identity	*W*,*a* *g* *e* ^2^,*f* *g* ^2^,*f* *h* *b* ^2^			
Norm 3	normal	identity	*W*,*a* *g* e*f *g*,*a* *g* e*f *h* *b*,*f* g*f *h* *b*,*m* *o* *t* *o* *r*3*f *h* *b*			
Norm 4	normal	identity	*W*,*a* *g* *e* ^2^,*f* *g* ^2^,*f* *h* *b* ^2^			
			*a* *g* e*f *g*,*a* *g* e*f *h* *b*,*f* g*f *h* *b*,*m* *o* *t* *o* *r*3*f *h* *b*			
Gam 1	gamma	log	*W*,*A*			
Gam 2	gamma	log	*W*,*a* *g* *e* ^2^,*f* *g* ^2^,*f* *h* *b* ^2^			
Gam 3	gamma	log	*W*,*a* *g* e*f *g*,*a* *g* e*f *h* *b*,*f* g*f *h* *b*,*m* *o* *t* *o* *r*3*f *h* *b*			
Gam 4	gamma	log	*W*,*a* *g* *e* ^2^,*f* *g* ^2^,*f* *h* *b* ^2^			
			*a* *g* e*f *g*,*a* *g* e*f *h* *b*,*f* g*f *h* *b*,*m* *o* *t* *o* *r*3*f *h* *b*			
Outcome estimation, regression						
Mortality				Costs		
Candidate	Error dist	Link fn	Linear pred	Error dist	Link fn	Linear pred
Linear	binomial	logit	*W*	normal	identity	*W*
Linear (costs)				gamma	log	*W*
Linear, int	binomial	logit	*W*,A*a *g* *e*,A*f *g*,A*f *h* *b*	gamma	log	*W*,A*a *g* *e*,A*f *g*,A*f *h* *b*
Quadr, int (1)	binomial	logit	*W*,*A*,*A* ^2^,A*a *g* *e*	gamma	log	*W*,*A*,*A* ^2^,A*a *g* *e*
			A*f *g*,A*f *h* *b*,*A* ^2^*a *g* *e*			A*f *g*,A*f *h* *b*,*A* ^2^*a *g* *e*
			*A* ^2^*f *g*,*A* ^2^*f *h* *b*			*A* ^2^*f *g*,*A* ^2^*f *h* *b*
Quadr, int (2)	Binomial	logit	*W*,*A*,*A* ^2^,A*a *g* *e*,A*f *g*	gamma	log	*W*,*A*,*A* ^2^,A*a *g* *e*,A*f *g*
			A*f *h* *b*,*A* ^2^*a *g* *e*,*A* ^2^*f *g*			A*f *h* *b*,*A* ^2^*a *g* *e*,*A* ^2^*f *g*
			*A* ^2^*f *h* *b*,A*a *g* *e* ^2^,A*f *g* ^2^			*A* ^2^*f *h* *b*,A*a *g* *e* ^2^,A*f *g* ^2^
			A*f *h* *b* ^2^,*a* *g* *e* ^2^,*f* *g* ^2^,*f* *h* *b* ^2^			A*f *h* *b* ^2^,*a* *g* *e* ^2^,*f* *g* ^2^,*f* *h* *b* ^2^
Fourth	binomial	logit	*W*,*A*,*A* ^2^,*A* ^3^,*A* ^4^	gamma	log	*W*,*A*,*A* ^2^,*A* ^3^,*A* ^4^
GAM	binomial	logit	splines of *W*,*A* (df=2,3)	normal	identity	splines of *W*,*A* (df=2,3)
Bayesian GLM	binomial	logit	*W*,*A*	normal	identity	*W*,*A*
Outcome estimation, GPS						
Mortality				Costs		
Linear	binomial	logit	*A*,*r*	normal	identity	*A*,*r*
Linear (costs)				gamma	log	*A*,*r*
Linear, int	binomial	logit	linear, *A*,*r*,A*r	gamma	log	*A*,*r*,A*r
Quadr, int (1)	binomial	logit	*A*,*r*,*A* ^2^,r*A,*A* ^2^*r	gamma	log	*A*,*r*,*A* ^2^,r*A,*A* ^2^*r
Quadr, int (2)	binomial	logit	*A*,*r*,*A* ^2^,r*A,*A* ^2^*r,*r* ^2^,*r* ^2^*A	gamma	log	*A*,*r*,*A* ^2^,r*A,*A* ^2^*r,*r* ^2^,*r* ^2^*A
Fourth	binomial	logit	*r*,*A* ^2^,*A* ^3^,*A* ^4^	gamma	log	*r*,*A* ^2^,*A* ^3^,*A* ^4^
GAM	binomial	logit	splines of *r*,*A* (df=2,3)	normal	identity	splines of *r*,*A* (df=2,3)
Bayesian GLM	binomial	logit	*A*,*r*	normal	identity	*A*,*r*

GPS,generalised propensity score; GAM, generalised additive models; GLM, generalised linear model. *W*: all covariates, *A* treatment variable, *r*: GPS, *fg*: glucose, *fhb*: haemoglobin, *m* *o* *t* *o* *r*3: motor score 3

For the the mortality outcome, we consider a range of logistic regression estimators; for the cost endpoint, we consider GLMs with gamma distribution and log link. For the regression approaches, we consider functional forms that include polynomials of the treatment variable and the continuous covariates, and interactions between these, in the linear predictor. Categorical covariates are introduced as linear terms without interactions. We follow a similar approach when specifying outcome models given the GPS and the treatment variable, for example, the specification with second‐order polynomials and interaction corresponds to the parametric specification suggested by Hirano and Imbens (2004) (see full specifications in Table 1).

Beyond these parametric models, we also consider further candidate algorithms to predict the outcome, which have performed well, in terms of bias reduction, in previous simulations (Kreif *et al.*, 2014) 4. We included generalised additive models (two and three degrees of freedom) to allow the expected outcome as a flexible, additive function of the covariates (Hastie, 2013). We included a Bayesian GLM, which includes main terms in the linear predictor, but uses non‐informative priors to estimate the coefficients (Gelman and Su, 2013). We specify a 10‐fold cross validation for the Super Learner (van der Laan and Dudoit, 2003).

#### 3.3.2 Estimation of uncertainty

We use the non‐parametric bootstrap (1000 samples) for statistical inference for the estimated dose–response function (between one and 24h) and the estimated marginal treatment effect (using one‐hour increments). In the absence of theoretical formulae for the asymptotic distribution of these parameters, the bootstrap allows to approximate the 95% confidence intervals (CIs) (Hirano and Imbens, 2004; Eliseeva *et al.*, 2013). By re‐estimating the GPS and the outcome model, the proposed bootstrap procedure takes into account the uncertainty in the estimation of the GPS, as well as in the selection of the outcome model. All computation is performed using the R platform (R Core Team, 2013).

#### 3.3.3 Assessing balance and overlap

We follow the blocking approach proposed by Hirano and Imbens (2004) and categorise the treatment variable by tertiles. Imbalance before GPS adjustment is assessed by comparing covariate means between groups of observations, which belong to the given treatment tertile, versus those in the remaining tertiles. We calculate the GPS‐adjusted version of these statistics, by first, evaluating the GPS for each observation, for the median treatment level in each tertile. For each tertile, we then categorise the estimated GPS, into five blocks, based on quantiles. Mean differences and corresponding *t*‐statistics are then computed within each GPS block, and their weighted mean is calculated, according to the number of observations in each block. Following recommendations of Kluve *et al.* (2012) and Flores *et al.* (2012), we use a similar logic for assessing the common support after estimating the GPS.

### 3.4 Results of the empirical example

#### 3.4.1 The estimated generalised propensity score, common support, balance

Table 2 describes the sample (*n*=488). The transfer time variable ranged from 1.83 to 23.7h (Supporting Information Figure 1). The Super Learner algorithm selected a mixture of the normal and gamma models for the GPS, assigning weights of 18, 32 and 16 and 3% to the normal model candidates, while a total of 32% to the gamma models.

**Table 2:** Descriptive statistics of baseline covariates and outcomes

**Variable**		*n* = 488
**Outcomes**		
Dead at 6months, n (%)	99	(20.3)
Sixmonths costs (*$*), mean (SD)	27 480	(29 741)
**Baseline covariates**		
IMPACT pred mort, mean (SD)	0.23	(0.17)
Age, mean (SD)	40.33	(17.51)
Major extr inj, *n* (%)	185	(37.9)
Severe GCS, *n* (%)	265	(54.3)
Motor score poor,*n* (%)	226	(46.3)
Any pupil unreactive, *n* (%)	76	(15.6)

SD, standard deviation; IMPACT pred mort, predicted mortality from IMPACT risk prediction model; extr inj, extracranial injury; GCS, Glasgow Coma Score.

The following variables had missing values for *n* observations: dead at 6months: *n* = 18, pupil unreactive: *n* = 41, motor score: *n* = 7.

When assessing the overlap in tertiles of the treatment variable, it appears that overlap is generally good, for the middle tertile, while there is a lack of overlap at values of the GPS close to zero, albeit only for 2.2% of observations (Supporting Information Figure 2).

Table 3 presents balance statistics as mean differences (*t*‐statistics) before and after adjustment with the GPS. Analogously to the assessment of common support, balance has been evaluated for the tertiles of the treatment variable, unadjusted and then adjusted for five blocks of the GPS. Patients with lower treatment levels were significantly younger, had a lower IMPACT‐predicted mortality score and were less likely to have a major extracranial injury. After adjustment, mean differences and *t*‐statistics were generally lower, although in the higher treatment categories, imbalance on age increased.

**Table 3:** Balance unadjusted and adjusted for the GPS: differences in means and *t*‐statistics for the equality of means

Variable	Unadjusted differences in means (*t*‐stat)			Adjusted differences in means (*t*‐stat)		
	tx cat [1.83–5.2]	tx cat [5.2–10.1]	tx cat [10.1–23.7]	tx cat [1.83–5.2]	tx cat [5.2–10.1]	tx cat [10.1–23.7]
IMPACT pred mort	−0.03 (1.99)	−0.00 (−0.04)	0.03 (1.92)	−0.01 (−0.91)	0.00 (0.18)	0.01 (0.81)
Age	−4.88 (−3.05)	3.87 (2.26)	0.99 (0.58)	−3.09 (−1.81)	4.22 (2.49)	−1.09 (−0.65)
Major extr inj	−0.08 (−1.69)	−0.02 (−0.35)	0.09 (1.99)	−0.02 (−0.32)	0.01 (0.12)	0.02 (0.47)
Severe GCS	0.07 (1.36)	−0.06 (−1.25)	−0.01 (−0.10)	0.03 (0.59)	−0.07 (−1.49)	0.04 (0.73)
Motor score poor	0.018 (0.38)	−0.069 (−1.45)	0.051 (1.06)	0.011 (0.23)	−0.068 (−1.42)	0.050 (1.00)
Any pupil unreactive	−0.01 (−0.33)	−0.03 (−0.92)	0.04 (1.18)	0.01 (0.15)	−0.03 (−0.76)	0.02 (0.61)

GPS, generalised propensity score; tx cat, treatment category; IMPACT pred mort, predicted mortality from IMPACT risk prediction model; GCS, Glasgow Coma Score. Differences in means reported, with *t*‐statistics for the equality of the mean in brackets. Each comparison contrast units in a given treatment category versus the other two treatment categories. There are 122, 123 and 123 patients in each group.

#### 3.4.2 Estimated dose–response functions

**Figure 1:**
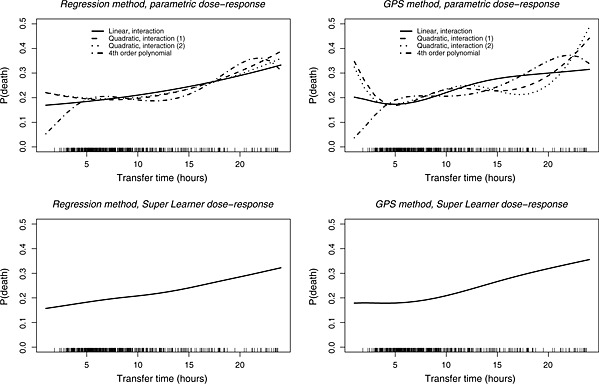
Dose–response functions of expected mortality at 6months, using regression and GPS, with parametric models and the Super Learner. The rug plots demonstrate the distribution of observed transfer times.GPS, generalised propensity score

We estimated dose–response functions for 6months mortality and costs, with different parametric specifications of the outcome model for the regression and the GPS approach (upper panels of Figure 1 and Supporting Information Figure 3). The plots suggest an increasing relationship in transfer time and expected mortality, however, some of the models estimated a non‐monotonic relationship. For example, when the GPS approach used a quadratic term of the treatment variable in the outcome model, as proposed by Hirano and Imbens (2004), the effect of increasing transfer time between 12 and 18h appears to reduce rather than increase expected mortality.

Table 4 presents the results from the cross‐validation performed by the Super Learner algorithm. The table displays the estimated MSEs of the candidate algorithms and the Super Learner estimator, alongside the weight each candidate received in the Super Learner selection. Amongst the parametric models, relatively simple outcome models, including linear terms only, provided the best fit in terms of cross‐validated MSE. For the mortality endpoint, none of the parametric models from the previous, exploratory analysis received a positive weight in the Super Learner. A combination of the Bayesian GLM prediction algorithm and the GAM received all weights for the regression, while for the GPS approach, the Bayesian GLM predictor was given full weight. For the cost outcome, the pre‐specified parametric models had positive weights with the Super Learner estimator, together with the Bayesian GLM.

**Table 4:** Cross validation results and Super Learner weights

Candidate predictor	Regression approach		GPS approach	
	MSE	Weight in Super Learner	MSE	Weight in Super Learner
	Mortality			
Linear	0.1472	0.00	0.1621	0.00
Linear, int	0.1503	0.00	0.1618	0.00
Quadratic, int (1)	0.1553	0.00	0.1631	0.00
Quadratic, int (2)	0.1576	0.00	0.1644	0.00
Fourth order	0.1484	0.00	0.1634	0.00
GAM (d.f.=2)	0.1470	0.31	0.1625	0.00
GAM (d.f.=3)	0.1487	0.00	0.1630	0.00
Bayesian GLM	0.1456	0.69	0.1617	1.00
Convex Super Learner	0.1481		0.1621	
	Costs			
Linear (normal)	853058	0.00	883591	0.00
Linear	855597	0.13	883507	0.83
Linear int	864127	0.14	889775	0.00
Quadr, int (1)	941058	0.00	895103	0.17
Quadr, int (2)	897010	0.20	912055	0.00
Fourth order	891008	0.00	901585	0.00
GAM (d.f.=2)	857542	0.00	887886	0.00
GAM (d.f.=3)	863129	0.00	894215	0.00
Bayesian GLM	852746	0.52	883579	0.00
Convex super learner	859842		888529	

GPS, generalised propensity score; MSE, mean squared error; d.f., degrees of freedom; GAM, generalised additive models; GLM, generalised linear model. MSE for costs in 1000 *$*.

The high weight given to the Bayesian GLM algorithm versus the main terms GLM reflects the use of non‐informative priors that shrink the estimated regression coefficients and result in slightly lower MSE for the Bayesian GLM. However, the estimated risks and dose–response functions are very similar to using main terms GLM only.

**Figure 2:**
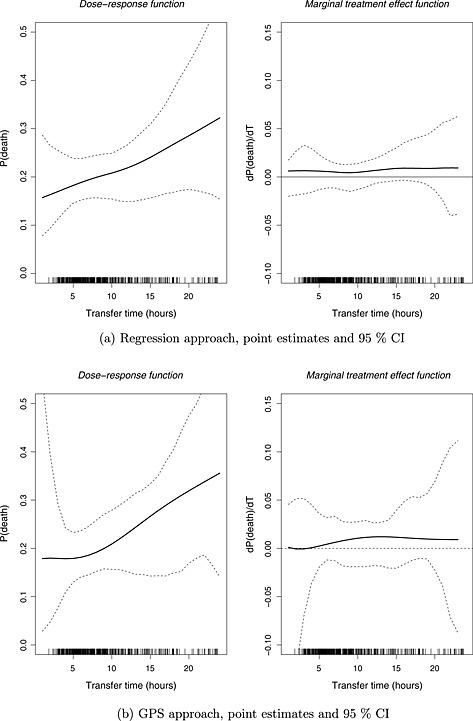
Dose–response function and marginal treatment effect function of expected mortality at 6months, using the Super Learner. (a) Regression approach, point estimates and 95% CI, and (b) GPS approach, point estimates and 95% CI. The rug plots demonstrate the distribution of observed transfer times. GPS, generalised propensity score; CI, confidence interval

The lower panel of Figure 1 presents the estimated dose–response functions with the Super Learner, for the mortality endpoint. Both the regression and GPS approaches suggest a monotonically increasing relationship. Figure 2 presents point estimates (95% CI) for the marginal treatment effect from the Super Learner estimators for mortality. The CIs for the marginal treatment effect function include zero, corresponding to a zero incremental effect of increasing transfer time on the expected outcomes. Hence, the null hypothesis, that increasing transfer time does not have an effect on expected 6months costs or mortality, cannot be rejected, at the 5% level of statistical significance. When comparing the CIs between the regression and the GPS approach, we find that the GPS approach reports wider intervals, especially for those treatment values where the empirical distribution of observed treatment is sparse. For the cost endpoint, the GPS approach suggests that expected costs initially decrease up to 7h and then increase with transfer time, but as for the mortality endpoint, the null hypothesis cannot be rejected (see Figures 3 and 4 in the Supporting Information).

## 4 Discussion

This paper provides a framework for estimating marginal causal effects of continuous treatments that does not require the model for the GPS or the outcome regression to be correctly specified. Instead, this paper proposes that a machine learning approach, the Super Learner, can be applied to the GPS method proposed by Hirano and Imbens (2004). We contrast this approach to parametric implementations of the GPS. The paper also compares the GPS approach to regression methods for estimating the dose–response function both with parametric implementation, and using the Super Learner.

The empirical example presented has typical characteristics of evaluations with moderate sample size, and where a plausible assumption about unconfoundedness requires adjustment for many binary and continuous covariates. We find that the regression and the GPS approaches are both sensitive to the choice of model specification for the outcome, and that the estimated dose–response curves differ by parametric specification. In this example, the Super Learner estimator assigned small weights to nonlinear models, which suggested that the non‐monotonic dose–response functions, estimated by some of the parametric models, were not supported by the data. With the Super Learner, the regression and the GPS approaches led to the same conclusion, namely that the marginal effect of increasing transfer time on mortality and cost was zero (at the 5% level of statistical significance).

In this example, 95% CIs were wider following the GPS versus the regression approach. This is expected, as estimators using the propensity score are usually less efficient than those from a correctly specified outcome model (Vansteelandt and Daniel, 2014). It should also be recognised that, as part of the RAIN study, an extensive systematic review of previous outcome regression models was undertaken (Harrison *et al.*, 2013), but there was little prior information on the form that the GPS may take. Hence, it was relatively more challenging to specify the covariate to treatment versus the covariate to outcome relationship.

This paper extends the GPS approach proposed by Hirano and Imbens (2004), for estimating the effects of continuous treatments and a related working paper by Bia *et al.* (2011), who propose flexible, spline‐based estimators for the outcome model. Even these approaches require subjective modelling choices, such as a pre‐fixed degree of polynomials and the number of knots in the spline, and so the Super Learner approach can further increase flexibility, by incorporating these estimators amongst the candidate predictors.

Our paper highlights a distinguishing feature of the Super Learner compared with other model selection approaches, in that it combines many estimators, by selecting a combination of predictions from alternative prediction algorithms. That is, the Super Learner aims to provide a better fit to the data than relying on any one pre‐specified prediction algorithm. To facilitate the consistent estimation of the dose–response function, the Super Learner should be required to consider a rich set of prediction algorithms (van der Laan *et al.*, 2007). In selecting the candidates, subject matter knowledge of the data‐generating process may be used, for example, in this paper, we included models with the gamma distribution and log link function to accommodate the skewed distribution of costs.

An important advantage of using the Super Learner is that it encourages transparency in model selection, a process that in applied research often remains informal and hidden. The Super Learner, as a prediction technique, only considers the predictive power of each algorithm, ignoring their internal workings. Therefore, the Super Learner does not provide model output, for example, estimated coefficients of individual covariates, that is directly interpretable.

In our sample, we found the MSE of the best single candidates (the ‘discrete’ Super Learner) was slightly lower than that of the convex Super Learner. Because the differences are small, choosing the best candidate estimators would provide similar dose–response functions to the convex Super Learner. In general, van der Laan *et al.* (2007) suggest that the convex Super Learner provides more stable estimates than the discrete Super Learner.

Our paper adds to the growing literature on the use of the Super Learner for causal inference (Gruber and van der Laan, 2010; Porter *et al.*, 2011; Kreif *et al.*, 2014; Neugebauer *et al.*, 2014; Pirracchio *et al.*, 2015b; Pirracchio *et al.*, 2015a), and more generally, to the implementations of machine learning methods in estimating treatment effects (Lee *et al.*, 2010; van der Laan and Rose, 2011; Austin, 2012). The setting of continuous treatments posed new challenges for the Super Learner framework, in having to represent the uncertainty in the estimator selection for both the GPS and the outcome regression, which this work has addressed with the non‐parametric bootstrap.

This paper has some limitations. First, each of the approaches relies on the assumption of no unmeasured confounding, specifically in the context of continuous treatments, the weak unconfoundedness assumption. This assumption requires that for any level of treatment, the probability of receiving this level is independent of the potential outcomes, conditional on covariates. In our empirical example, this assumption required that factors that cause delays in transfer, and are also prognostic of the outcomes, are controlled for. We used potential confounders from a previously validated risk prediction model, as well as clinical opinion, to pre‐specify a set of variables. However, the possibility for unobserved confounding remains, for example, because the covariates are measured at the time of hospital presentation, so subsequent changes in patients' prognosis, which might cause delays in transfer and affect 6‐month outcome, are unmeasured. In the absence of appropriate instrumental variables, the effects of unobserved confounding could be examined by extending sensitivity analysis methods to the context of continuous treatments (Rosenbaum, 1987).

Second, in this study, covariate balance following adjustment with the GPS did not improve for all variables. An alternative loss function for the Super Learner could explicitly consider a metric that takes into account the balance achieved. Methodological advances have been made in using machine learning methods (Lee *et al.*, 2010; Zhu *et al.*, 2014; McCaffrey *et al.*, 2013) to estimate the propensity score, for binary (Lee *et al.*, 2010) as well as continuous (Zhu *et al.*, 2014) and multi‐valued (McCaffrey *et al.*, 2013; Imai and Ratkovic, 2014; Wyss *et al.*, 2014) treatments, directly targeting balance. All of these approaches still require subjective choices of the appropriate balance measures, and the prioritisation of confounders (Stuart, 2010). Third, in using a single imputed dataset, the estimates of the treatment effects ignore the uncertainty in the estimation of the imputation model used to handle missing data.

This work provokes areas of further research. Future simulation studies could examine the sensitivity of the dose–response curve to the misspecification of the GPS and investigate how this misspecification can be assessed by evaluating the balancing property of the GPS.

While the regression and GPS approach lead to similar conclusions in our empirical example, more generally, a criterion for choosing between these approaches is required. An approach for choosing between dose–response curves was recently proposed by Díaz and van der Laan (2013), building on the principles of Super Learning.

## Supporting information

Supporting info item

Supporting info item

## Footnotes

1 Our outcome models are a conditional mean of a continuous endpoint (costs) and a conditional probability (mortality). The latter may be expressed as the conditional expectation of a binary variable, therefore, the MSE is an appropriate loss function. Note that this is an arbitrary choice, as other loss functions such as the negative log‐likelihood of a binary variable are also appropriate. Our choice allows us to use existing software such as the superlearner package in R.

2 To unify notation, we use *Q*(*w*) for the outcome model both as a function as covariates and treatment and as a function of the GPS and treatment, which was denoted with *β*(*t*,*r*) before.

3 In other words, the data did not follow a simple hierarchical structure whereby patients within a particular geographic region were nested within particular critical units, from which patients were transferred to specific neuroscience centres.

4 In order to control for all the pre‐specified potential confounders, we excluded candidate algorithms which perform variable selection.
