# Supplementary material for: Evaluation of the Effect of a Continuous Treatment: A Machine Learning Approach with an Application to Treatment for Traumatic Brain Injury
Source: Health Econ. 2015 Jun 8;24(9):1213–28. doi: 10.1002/hec.3189 (PMC4744663; doi:10.1002/hec.3189)
Supplement: Supplementary file 1 — Supporting info item [file HEC-24-1213-s001.pdf]

Online appendix to the paper: "Evaluation of the effect of a continuous treatment: a machine learning approach with an application to treatment for traumatic brain injury"

March 13, 2015

1 Supplementary figures

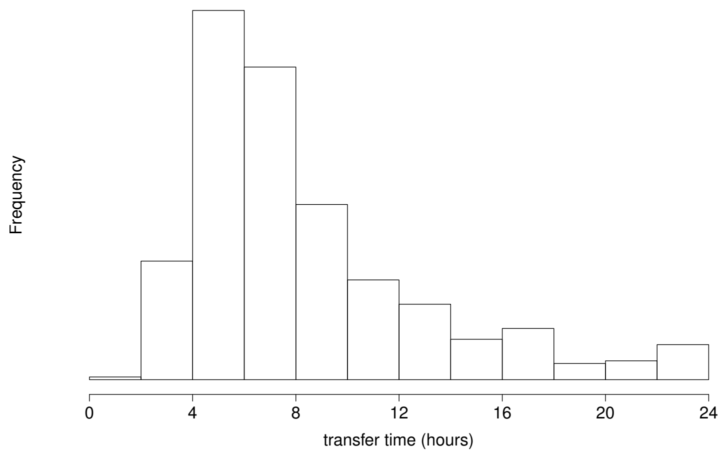

(a) Transfer time (hours)

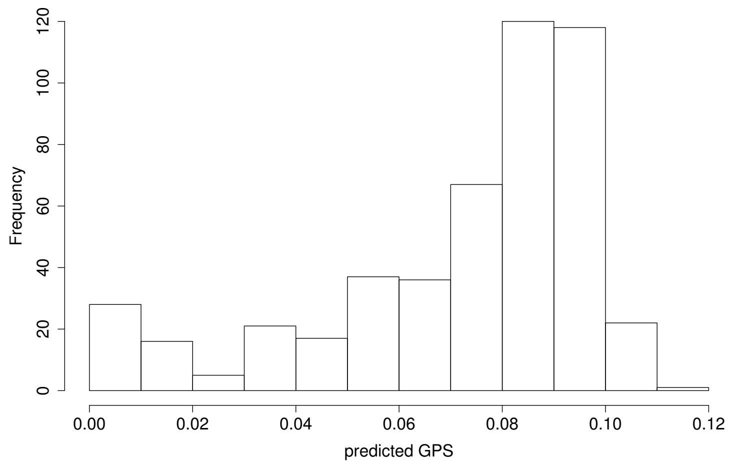

(b) Estimated GPS

Figure 1: The distribution of (a) transfer time (hours) and (b) the estimated GPS

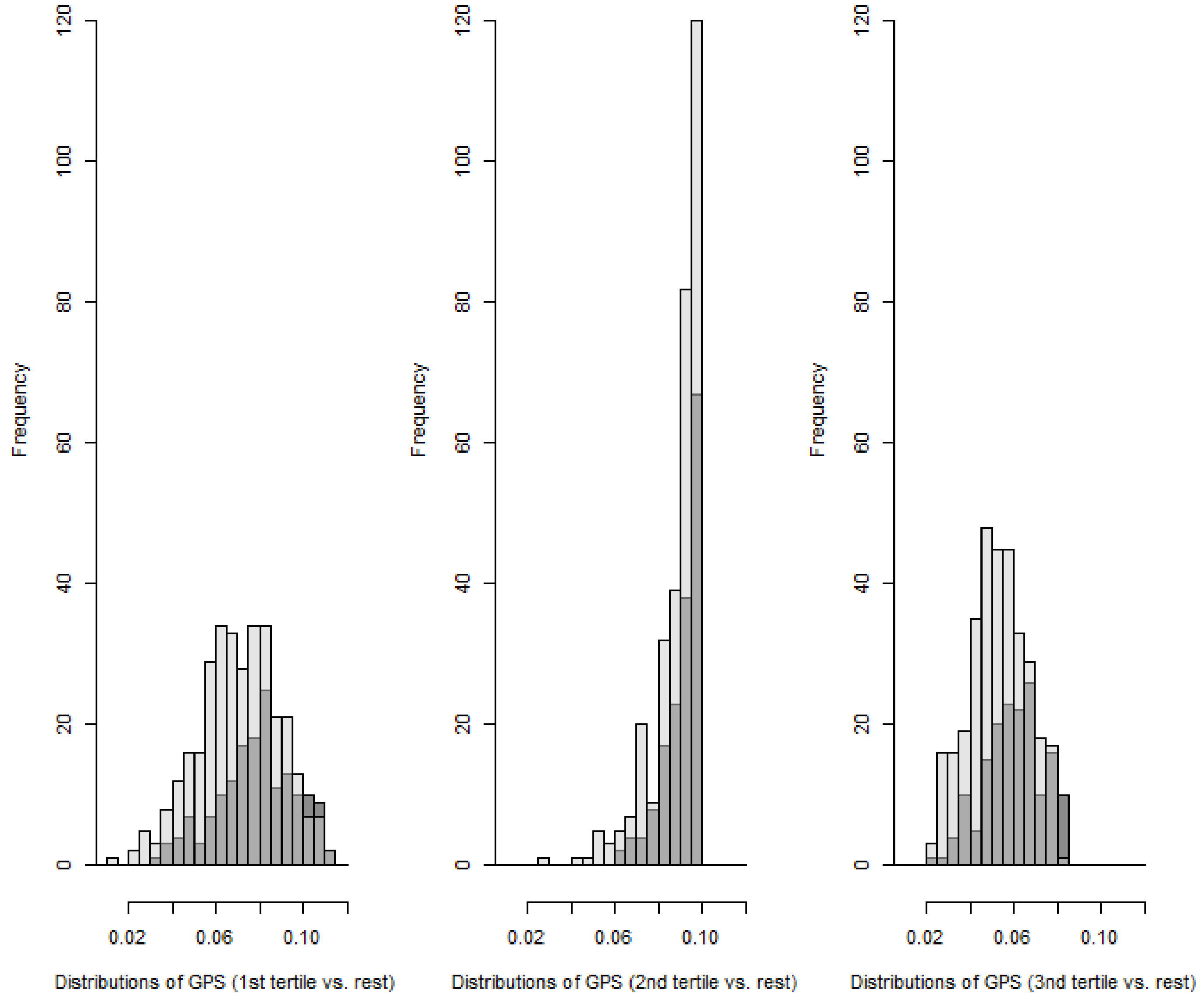

Overlap was evaluated in the treatment tertiles: transfer time between 1.83 – 5.2 hours (n=122), 5.2 – 10.1 hours (n=123) and between 10.1 – 23.7 hours (n=123). The three graphs compare the distribution of GPS evaluated at the medians of treatment values of the three groups, 4.66, 6.93 and 12.25. The dark grey histograms show the distribution of the GPS for those who received the treatment level of that category. The light grey histogram shows the GPS evaluated at the same level, but for those who received treatment of different levels.

Figure 2: Overlap, based on the GPS estimated at medians of tertiles of the transfer time distribution

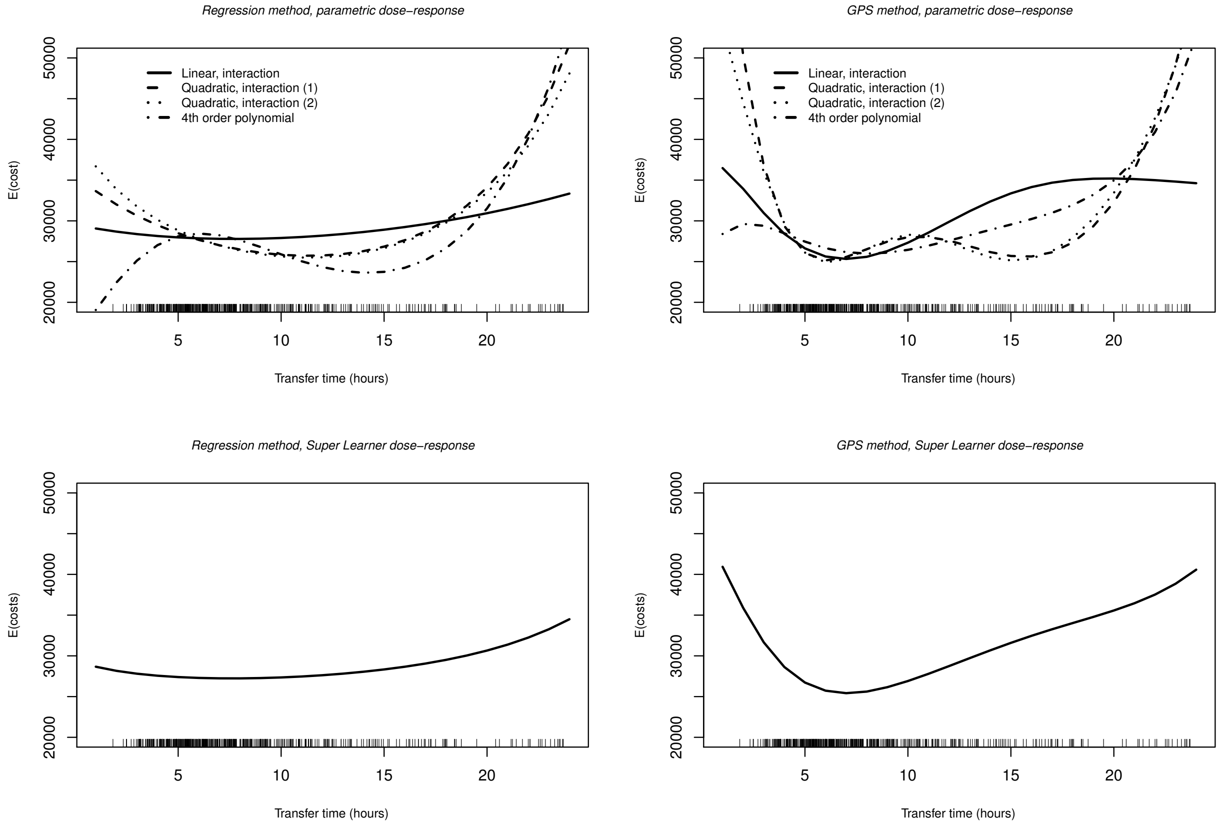

The rug plots demonstrate the distribution of observed transfer times.

Figure 3: Dose-response functions of expected costs at six months, using regression and GPS, with parametric models and the Super Learner

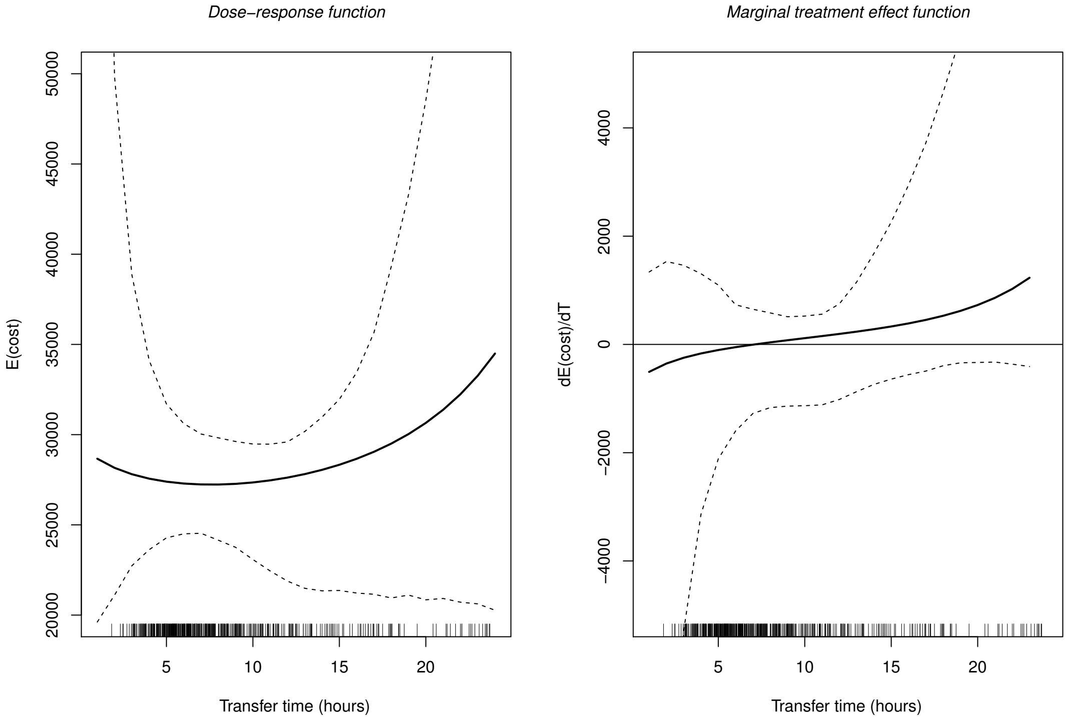

(a) Regression approach, point estimates and 95 % CI

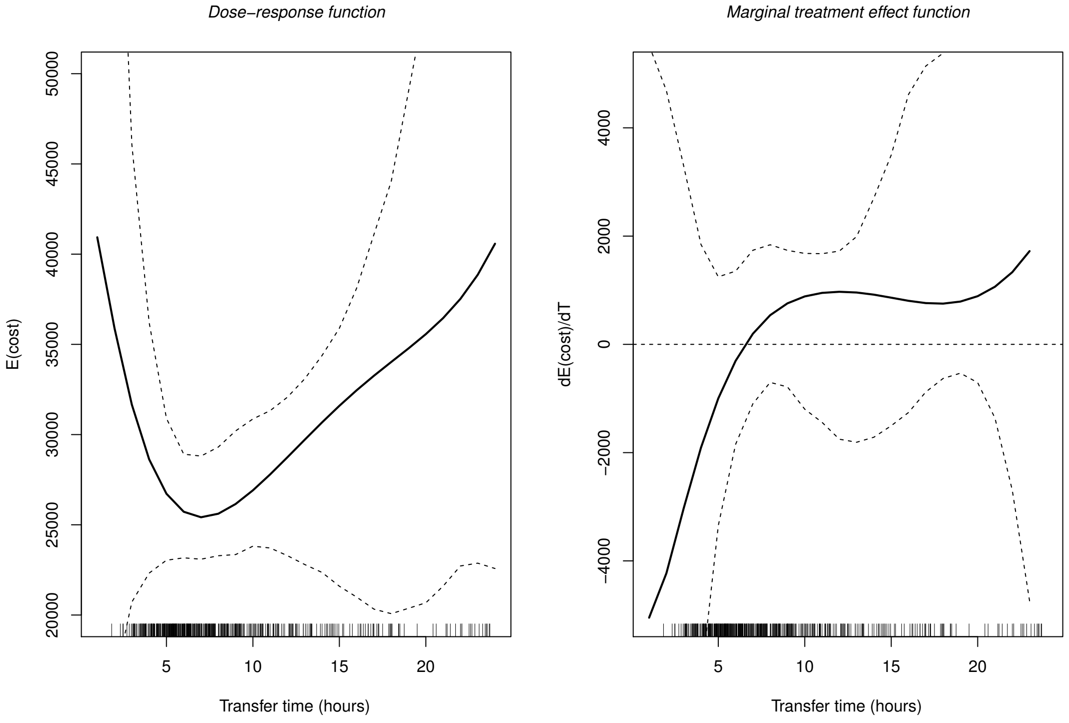

(b) GPS approach, point estimates and 95 % CI

The rug plots demonstrate the distribution of observed transfer times.

Figure 4: Dose-response function and marginal treatment effect function of expected costs at six months, using the Super Learner
